# IDENTIFYING THE PATTERNS OF CAREGIVING DEMANDS: A LATENT CLASS ANALYSIS APPROACH

**DOI:** 10.1093/geroni/igad104.2885

**Published:** 2023-12-21

**Authors:** Sung Hyun Ko, Yeonjung Lee, Alex Bierman

**Affiliations:** Chung-Ang University, Seoul, Republic of Korea; Chung-Ang University, Seoul, Republic of Korea; University of Calgary, Calgary, Alberta, Canada

## Abstract

Caregiving demands comprise the care tasks being performed or assistance being provided. Previous studies have assessed the care demands as intensity and types of care provided using various measures such as the number of care tasks provided, the hours of care provided, the number of ADL/IADL they assisted with, and the number of people assisted. However, most caregiving literature has treated caregiving demands as one-dimensional rather than considering the multi-dimensional attributes of care demands. This study examines distinct patterns of caregiving demands, each formed by varied combinations of the hours spent providing care a week, how often care was provided, the location care provided, and whether care was provided as a primary caregiver. Based on the 2022 Caregiving, Aging, and Financial Experiences (CAFE) Study data, the latent class and regression analysis were conducted on a nationally representative sample of Canadian informal caregivers. We identified four distinct patterns of caregiving demands: excessive, moderate, manageable, and minor. Excessive and moderate patterns share the characteristic of providing in-home care as primary caregivers, but the excessive group has higher burden levels than the moderate pattern. Manageable and minor patterns show low levels of burden as secondary caregivers, but the minor group does not provide in-home care. These patterns differ in caregivers’ sociodemographic characteristics (caregiver age, gender, minority, education, living with a partner, working status, and relationship to a recipient). The findings provide the basis for further understanding how the multi-dimensional layers of care demand conjointly shape the patterns of caregiving demands.

